# The association between screen time and cardiometabolic risk in young children

**DOI:** 10.1186/s12966-020-00943-6

**Published:** 2020-04-29

**Authors:** Harunya Sivanesan, Leigh M. Vanderloo, Charles D. G. Keown-Stoneman, Patricia C. Parkin, Jonathon L. Maguire, Catherine S. Birken, on behalf of the TARGet Kids! Collaboration

**Affiliations:** 1 Master of Public Health, Epidemiology, University of Toronto, Toronto, Canada; 2 The Hospital for Sick Children Research Institute, Child Health and Evaluative Sciences, SickKids Research Institute, Toronto, ON Canada; 3 Applied Health Research Centre, Li Ka Shing Knowledge Institute of St. Michael’s Hospital, Toronto, Ontario Canada; 4 Division of Biostatistics, Dalla Lana School of Public Health, University of Toronto, Toronto, Ontario Canada; 5 Division of Paediatric Medicine, Hospital for Sick Children, Toronto, Ontario Canada; 6 Child Health and Evaluative Sciences, SickKids Research Institute, Toronto, ON Canada; 7 Institute of Health Policy, Management and Evaluation, University of Toronto, Toronto, Ontario Canada; 8 Department of Pediatrics, Faculty of Medicine, University of Toronto, Toronto, Ontario Canada; 9 Department of Pediatrics, St. Michael’s Hospital, Toronto, Ontario Canada; 10 The Centre for Urban Health Solutions, Li Ka Shing Knowledge Institute of St. Michael’s Hospital, Toronto, ON Canada; 11 Joannah & Brian Lawson Centre for Child Nutrition, Department of Nutritional Sciences, University of Toronto, Toronto, ON Canada

**Keywords:** Sedentary behavior, Screen time, Cardiometabolic health, Triglycerides, Cholesterol, Waist circumference, Systolic blood pressure, Physical activity

## Abstract

**Objectives:**

While studies exist on the association between screen time and cardiometabolic risk among adolescents, research examining the effect of screen time on cardiometabolic risk in young children is lacking. The primary objective of this study was to examine the association between daily screen time and cardiometabolic risk (CMR) [sum of age- and sex-standardized z-scores of systolic blood pressure (SBP), glucose, log-triglycerides, waist circumference (WC), and negative high-density lipoprotein (HDL) cholesterol divided by the square root of five] in young children. Secondary objectives included examining individual CMR risk factors, including waist-to-height ratio and non high-density lipoprotein (non-HDL) cholesterol, as well as the individual cut-offs of these risk factors. Additional analyses include examining the association between screen time and CMR by handheld/non-handheld devices.

**Methods:**

A study was conducted among young children 3 to 6 years from the TARGet Kids! practice-based research network in Toronto and Montreal, Canada. Children with one or more measures of screen time and CMR were included in this study. Generalized estimating equation (GEE) multivariable linear regressions and multivariable logistic regressions, using published cut-offs, were conducted to evaluate these associations.

**Results:**

Data from 1317 children [mean age 52 months (SD = 13.36), 44.34% female] were included for analyses. There was no evidence of associations between screen time and total CMR score or individual risk factors (*p* > 0.05) after adjusting for confounders. A statistically significant, but small association between daily screen time and non-HDL cholesterol was found (B = 0.046; CI = [0.017 to 0.075]; *p* = 0.002.

**Conclusions:**

Though no relationship was reported between daily screen time and the majority of CMR factors in early childhood, there was an association between daily screen time and non-HDL cholesterol. As the relationship between daily screen time and CMR factors may not be apparent in early childhood, studies to evaluate longer-term cardiometabolic effects of screen time are needed. Although there is an evidence-based rationale to reduce screen time in early childhood, prevention of cardiometabolic risk may not be the primary driver.

## Introduction

Young children, adolescents, and adults are spending a substantial amount of time in sedentary behavior, including screen time, which may increase the risk of cardiometabolic disease [1, 2]. Screen time is defined as the time spent engaging in screen-related activities such as TV, DVD/video, smart phones, tablets and playing computer or video games [3]. According to the Canadian 24-Hour Movement Guidelines, children under the age of 2 years are not recommended to engage in any screen time [4], children between 2 and 5 years are recommended to engage in less than an hour of screen use per day [4], while children over the age of 5 years are recommended to engage in less than 2 h of recreational screen use per day [4]. Similarly, the World Health Organization (WHO) guidelines for screen time suggests that children under 1 year of age are not recommended to engage in any screen time, children 1 to 4 years are recommended to engage in no more than 1 h per day [5]. These align with guidelines from other countries, such as in Australia and New Zealand [6, 7]. Studies show that more than 50 % of Canadian children aged 4 to 7 years exceed the screen time recommendations [4, 8–10]. Health outcomes related to screen time have been examined in several studies [8, 11–26] and a systematic review of observational studies (*n* = 48) demonstrated that increased screen time was associated with increased weight gain and obesity [16].

Mechanisms to explain this association include increased intake of highly advertised foods, reduced ability to regulate food intake during screen time [27–30], and displacement of physical activity [27, 31]. While research is lacking on the effect of screen time on cardiometabolic risk (CMR) factors like systolic blood pressure, glucose, waist circumference and non-HDL cholesterol in early childhood, several studies have been conducted in adolescents and early adulthood, with varying results [8, 11, 12, 19–26]. Many studies have cited dietary intake as one of the primary mechanisms underlying the association between screen time and cardiometabolic risk [28, 32, 33]. For instance, a study has found that screen time leads to impaired satiety signals through the mental-reward system, and hence, increasing food intake [32]. Limitations of published work to date include cross-sectional study designs [8, 12, 19, 21–23, 26], lack of inclusion of screen time from handheld devices such as tablets or smartphones, [34] and measurements of screen time on weekdays only. Though most studies to date have been cross-sectional in nature, a recent longitudinal cohort study found that there were no associations between screen time and blood pressure or BMI, but positive associations with fatness measured by skinfold thickness [35]. There is also lack of studies focusing on early childhood; most have been conducted with adolescents and young adults [11, 12, 21, 23, 24]. One small study in Brazil explored the relationship between TV time and blood pressure in young children [25], with no association identified.

As such, the primary objective of this study was to examine the association between daily screen time and total CMR score in children 3 to 6 years. Secondary objectives included examining individual CMR risk factors (systolic blood pressure (SBP), glucose, log-triglycerides, waist circumference (WC), and negative high-density lipoprotein (HDL) cholesterol), including waist-to-height ratio and non-HDL cholesterol, as well as individual published cut-offs for these risk factors. This study also examines the association between screen time and CMR factors by handheld vs. non-handheld devices.

## Methods

### Study design and population

A study with repeated measures was conducted with participants from The Applied Research Group for Kids (TARGet Kids!) between July 2011 and May 2018. TARGet Kids! is a primary care practice-based research network (www.targetkids.ca) in Toronto and Montreal, Canada [36, 37]. Children under the age of 6 were recruited during their well-child physician visits from 11 primary care practices, between July 2008 and May 2017. Children are invited to participate in their following annual well-child physician visit, and this data were also included.

### Exposure variable

The primary exposure was daily screen time assessed using the parent-reported *Nutrition and Health Questionnaire* (NHQ) [36], a survey derived from the Canadian Community Health Survey [38]. Only one parent was required to complete the questionnaire at each time point and were primarily the mothers. Participants were asked "how much time did you spend ‘watching TV’, ‘DVD/videos’, ‘computer/laptop’, ‘playing video games’, or using handheld devices. Data on the type of screen device (TV, DVD/videos, computer/laptop, videogame), and screen time duration on a typical weekday and weekend day were included.

### Outcome variables

Height, weight and WC were measured by trained research staff during visits to the child’s primary care physician. Weight was measured using a precision digital scale (SECA, Germany), standing height was measured using a stadiometer (SECA, Germany) and the WC was measured using standardized protocols with a measuring tape [39]. Height was measured to the nearest 0.1 cm while weight is measured to the nearest 0.1 kg. As per the TargetKids protocol, following the calculation of BMI z-scores, the age and sex-standardized BMI z-score were calculated according to the WHO growth standards using the igrowup package [40]. The primary outcome, total CMR score, was a continuous measure quantified as the sum of z-scores from glucose, WC, SBP, negative HDL cholesterol, and log-triglycerides. The sum of these z-scores was then divided by the square root of five [31, 37, 41, 42]. Secondary outcomes were the individual measures included in the total CMR score, and additional CMR measures shown in the literature to be associated with poor CMR, including glucose, WC, SBP, HDL, log-triglyceride [37], waist-to-height ratio and non-HDL cholesterol [8, 19, 22, 23, 37, 43]. Glucose, HDL cholesterol, non-HDL cholesterol and triglycerides were collected via non-fasting blood samples (4–7 ml) drawn by trained pediatric phlebotomists. Given the young age of the participants, collecting fasted blood samples is difficult; and previous studies have shown that duration of fasting has a small impact on glucose and triglycerides in young children [44]. Glucose was measured using an enzymatic reference method with hexokinase; triglycerides, HDL cholesterol, and non-HDL cholesterol were measured using enzymatic colorimetric on the Roche Modular platform (Roche Diagnostics, Laval, Canada) [36]. The data used for CMR score were collected at the same time as for screen use, with some participants contributing repeated measures of both the CMR score and screen use (with 18.7% having at least 2 measures of exposure and outcome, and 5.0% having 3 measures or more of exposure and outcome).

### Covariates

The covariates (child age, child sex, maternal ethnicity, parental income, eating while watching TV, sugar-sweetened beverages (SSB), child’s BMI, child’s physical activity, family history of CVD, and fasting time [2, 8, 9, 12, 20–25] were collected through the NHQ as per the TARGet Kids! protocol [36] and were determined as co-variates a priori from the literature review. The data for the covariates (BMI, eating while watching TV, SSB, physical activity level) were collected at the same time as for screen time during their well-child visits. Parents reported their history of cardiometabolic disease (heart disease, hypertension, high cholesterol, and/or diabetes), as well as child age, sex, income, and maternal ethnicity at each of their well-child visits. Eating while watching TV was measured by asking “On a typical weekday/weekend day, which meals (breakfast, lunch, dinner, snack) did your child eat in a room with a screen device on (e.g., television, computer, handheld device)”. Unstructured free play outside of school was measured by asking, “Aside from time in daycare and school, on a typical weekday; how much time does your child spend outside in unstructured free play”. For sugar-sweetened beverages, the following question was asked as “how many cups of each (100% juice: apple, orange, etc; sweetened drinks: Sunny D, Kool aid, etc; soda/pop) your child drinks in a typical day” (see Table 1). For maternal ethnicity, the variable was self-declared by parent(s) and was categorized as ‘European’, ‘East Asian’, ‘South/Southeast Asian’, ‘Other’ (Arab, African, Latin American, Mixed Ethnicity). Family income was categorized as “$ < 30,000”, “$30000 to $79000”, “$80000 to $149000”, “$150,000+”. Family history of CVD was categorized as “Yes” or “No” (see Table 1).

**Table 1:** Baseline characteristics of study sample (*n* = 1317)

Variable	N with % or Mean +/− SD
Child age (months)	52.26 (36.00, 84.00)
Child sex	
Female	584 (44.34%)
Male	733 (55.66%)
Maternal ethnicity	
East Asian	84 (6.38%)
European	742 (56.34%)
South/Southeast Asian	169 (12.83%)
Other	192 (14.58%)
Family annual income	
< $30,000	72 (5.47%)
$30,000 to $79,999	454 (34.47%)
$80,000 to $149,000	255 (19.36%)
$150,000 +	536 (40.70%)
Family history of CVD	
Yes	184 (13.98%)
No	904 (68.64%)
Fasting time (hour)	2.41 (2.60)
Total screen time (h/week)	6.49 (5.13)
Glucose (mmol/L)	4.58 (0.64)
SBP (mmHg)	88.28 (8.15)
Log triglycerides (mmol/L)	−0.04 (0.51)
Waist-to-height ratio	0.50 (0.04)
HDL (mmol/L)	1.41 (0.35)
WC (cm)	52.76 (4.21)
Non-HDL (mmol/L)	2.57 (0.65)
Child’s BMI (zBMI)	0.21 (1.04)
Total cardiometabolic risk	
(CMR) score (z score)	−0.05 (1.11)
Physical activity (mins)	56.17 (55.00)
Eating while watching TV (number of meals)	1.73 (1.97)
SSB (number of cups)	0.86 (1.82)

*SBP* systolic blood pressure, *HDL* high-density lipoprotein cholesterol, *BMI* body mass index, *WC* waist circumference, *CVD* cardiovascular disease, *SSB* sugar-sweetened beverages. Continuous variables are shown as median, quartiles, and mean/standard deviation. Categorical variables are shown as number of subjects and percentages

### Inclusion and exclusion criteria

**Fig. 1:**
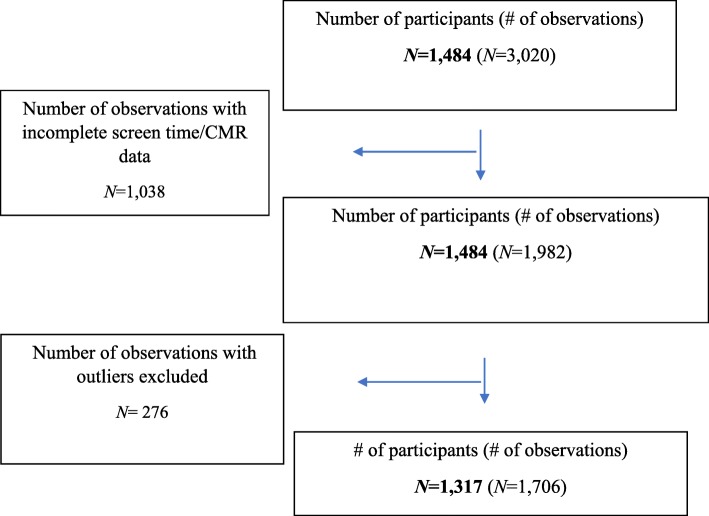
Flowchart describing the sample size and final number of observations in the dataset

Children were excluded from participation if, at recruitment in TARGet Kids!, they had health conditions affecting growth (e.g., failure to thrive, cystic fibrosis), severe developmental delays, chronic conditions (except asthma), or whose families were not able to communicate in English. For this study we included children between the ages of 3 to 6 years, who had parent-reported screen data (TV, DVD/video, videogame, computer, or handheld devices) and measures of CMR, including glucose, WC, SBP, HDL cholesterol, triglycerides, waist-to-height ratio, and non-HDL cholesterol, collected at least once. Any children who were missing screen time data or any of the CMR components were excluded from the analysis (Fig. 1). The Research Ethics Boards at the Hospital for Sick Children and St. Michael’s Hospital approved the study protocol and informed consent was obtained from the parents of children who participated in this cohort study.

### Statistical analyses

All data cleaning and data analyses were performed using the statistical software R (version 3.4.3) [45]. Outliers were removed based on biological implausibility for the CMR components [46–51]. For CMR measures like systolic blood pressure, we used the literature to identify the thresholds (< 0 or > 200 mmHg) [52]. For the other CMR components (BMI, WC, glucose, HDL, non-HDL), we excluded all the values that are outside range of − 6 and 6 for z-scores of WC, glucose, HDL, and non-HDL, as well as excluding values in the outside range of − 5 and 5 for zBMI zBMI [53]. Screen time was summed as the time spent in front of TV, DVD, computer, videogame, and handheld devices on both a weekday (× 5) and a weekend day (× 2). The z-score of HDL cholesterol was multiplied by − 1 as it is inversely related to total CMR score. All continuous outcome variables were verified for normality and any necessary transformations were performed. A logarithm transformation was performed on triglycerides to normalize the skewed distribution.

Children who had complete data for at least one type of screen time (TV, computer/laptop, DVD/video, videogames, or handheld devices) were retained for analyses. The percentage of missing values in the covariates variables in our sample was low and ranged from 0.2 to 6.5%. Multiple imputation analysis was performed using chained equations to impute missing values, with 15 datasets for each model [54]. Descriptive statistics (mean ± SD; percentage) were obtained for the main outcome, main exposure, and the covariates. Generalized estimating equation (GEE) univariable linear regressions were used to assess the unadjusted association between screen time and total CMR score, and each of the components of the total CMR score (SBP, log-triglycerides, glucose, HDL, and WC), non-HDL, and waist-to-height ratio. An adjusted GEE multivariable linear regression was performed after adjusting for the prespecified, clinically relevant confounders (child age, child sex, maternal ethnicity, parental income, eating while watching TV, SSB, physical activity, family history of CVD, fasting time, and child zBMI). Since child zBMI may be an intermediate variable rather than a confouder, [55, 56] we also performed analyses without the inclusion of child zBMI. Summaries of the results of GEE models were used to calculate the effect sizes of these associations, confidence intervals, and to test for significance of these relationships (see Table 3). We have also performed adjusted and unadjusted univariable/multivariable linear regression to look at the association between handheld and non-handheld screen time with total CMR score, and CMR factors. This study has multiple measures of both screen time and CMR which is accounted for in the model.

The cut points for high-risk were determined using existing standards for children [57] or using the 90th percentile in the absence of defined criteria. The cut-points for high-risk of the cardiovascular components were defined as follows: glucose (>90th percentile), systolic blood pressure (>90th percentile), triglycerides (0.84 mmol/L), waist-to-height ratio (>90th percentile), HDL cholesterol (< 1.17 mmol/L), non-HDL cholesterol (> 3.11 mmol/L), and WC (>90th percentile). Residual analyses were performed for both linear and logistic regression to verify assumptions of the regression models [58–60]. Sensitivity analyses were performed using complete cases reporting all screen time variables. Interactions were assessed for screen time*age and screen time*sex in both the linear and the logistic regression models. The variance inflation factor (VIF) was used to determine the presence of co-linearity among the multiple variables in both the linear and logistic regression models [61]. For all analyses, statistical significance was set at a value of *p* < 0.05. Sensitivity analyses were conducted to explore the role of three covariates (sugar sweetened beverages, eating while watching TV, physical activity) as potential mediators in this analyses.

## Results

In total, the study included a total of 1317 children with 1706 observations (see Fig. 1) with repeated measures of both screen time and cardiometabolic risk collected at the same time. The description of baseline characteristics is shown on Table 1. The sample consisted of 44.3% females and the children were on average 52 months old (*SD* = 12.93).

### Screen time

Table 2 shows the mean and standard deviation of screen time by sex, handheld vs. non-handheld devices, and weekday and weekend day. Males generally spent more time in front of screens with an average of 1.5 h per day, compared to females, with 1.4 h per day. Overall, the average time spent in front of screens on a typical weekend day (1.9 h per day), was higher, compared to a typical weekday with 1.3 h per day. The mean screen time for non-handheld devices was 1.3 h per day, compared to handheld devices, with 0.2 h per day. Children spent the most time in front of a TV (1.1 h per day) compared to other types of screens, such as DVD, for example (0.4 h per day; see Table 2). Among those 3 to 4 years, 20% of children met screen time guidelines of ≤ 1 h/day with similar percentage of boys and girls meeting the guidelines. In children over the age of 5 years, 38% met the screen time guideline of ≤2 h/day.

**Table 2:** Screen time (h/day) by child sex, type of screen (TV, DVD, videogame, computer, handheld), and by weekday/weekend day

Variables	Mean +/− SD
**Child sex**	
Male	1.5 ± 1.2
Female	1.4 ± 1.8
**Weekday screen use**^**a**^	1.3 ± 1.4
TV	0.8 ± 1.0
DVD	0.2 ± 0.5
Videogame	0.1 ± 0.2
Computer	0.12 ± 0.3
Handheld	0.2 ± 0.4
**Weekend day screen use**^**b**^	1.9 ± 1.4
TV	1.1 ± 1.1
DVD	0.4 ± 0.7
Videogame	0.1 ± 0.3
Computer	0.1 ± 0.3
Handheld	0.3 ± 0.5
**Type of device**	
Handheld^c^	0.2 ± 0.6
Non-handhled^d^	1.3 ± 0.8

^a^Weekday screen time use includes the sum of TV, DVD, computer, videogame, and handheld use from a typical weekday

^b^Weekend day screen time use includes the sum of TV, DVD, computer, videogame, and handheld use from a typical weekend day

^c^Handheld device use includes the sum of handheld use on a typical weekday and a typical weekend day

^d^Non-handheld device use includes the sum of TV, DVD, computer, and videogame use on a typical weekday and a typical weekend day

### Screen time and Cardiometabolic risk

No significant associations were found between total screen time and total CMR score as seen in the adjusted model (B = − 0.028; CI = [− 0.085; 0.030]; *p* = 0.030), in all models. In the secondary analysis, after adjustments, no association was found between screen time and individual CMR factors, including glucose, SBP, log triglycerides, WC, and waist-to-height ratio (*p* > 0.05). Total screen time was positively associated with mean non-HDL^cholesterol^ in the unadjusted (B = 0.039; CI = [0.015 to 0.063]; *p* = 0.001) and adjusted models (B = 0.046; CI = [0.017 to 0.075]; *p* = 0.002). After adjusting for covariates, every additional 60 min spent with screen time was associated with a 0.046 mmol/L higher mean blood levels of non-HDL cholesterol. No significant associations were found between handheld screen time and CMR (B = − 0.136; CI = [− 0.380; 0.107]; *p* = 0.264) (Supplemental Table 1) or between non-handheld devices and total CMR score (B = − 0.014; CI = [− 0.077; 0.048]; *p =* 0.657) (Supplemental Table 2). The associations between handheld and non-handheld were also non-significant for glucose, HDL, log triglycerides, WC, and waist-to-height ratio (*p* > 0.05). However, non-handheld devices were positively associated with mean non-HDL cholesterol in both the unadjusted (B = 0.036; CI = [0.011 to 0.061]; *p* = 0.005) and adjusted models (B = 0.039; CI = [0.010 to 0.069]; *p* = 0.008). Though we have identified evidence of an interaction between sex and screentime for SBP (*p* = 0.046), the associations between screen time and SBP were not individually significant for males (B = 0.161; CI = [− 0.252, 0.573]; *p* = 0.445) or females (B = − 0.522; CI = [− 1.142, 0.098]; *p* = 0.099) (Table 3). Similarly, no significant associations were found between handheld devices and SBP in males (B = 0.124; CI = [− 1.102, 1.351]; *p* = 0.842), and females (B = − 0.610; CI = [− 1.974, 0.754]; *p* = 0.381) (Supplemental Table 1). This was also found for non-handheld devices and SBP in males (B = 0.166; CI = [− 0.292, 0.624]; *p* = 0.477), and females (B = − 0.558; CI = [− 1.220, 0.104]; *p* = 0.099) (Supplemental Table 2), Of note, in the adjusted model where zBMI was not included, each 1 h increase in screen time was associated with an increase in SBP by 0.48 mmhg (95% CI 0.073 = − 0.89).

**Table 3:**
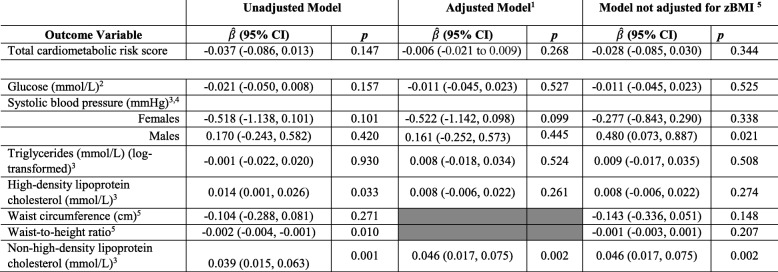
Linear GEE regression model for association between screen time (hr/day) and total cardiometabolic risk score and individuals risk factors, *n* = 1317

^1^Adjusted model includes adjustment for child age, child sex, maternal ethnicity, parental income, child’s BMI, family history of CVD, physical activity, sugar-sweetened beverages, and eating while watching TV

^2^Partially adjusted model includes adjustment in adjusted models, except for child’s BMI

^3^Model with glucose, systolic blood pressure, triglycerides, high-density lipoprotein cholesterol, and non-high-density lipoprotein cholesterol were adjusted for fasting time

^4^Models with systolic blood pressure is further adjusted for height, and include interactions for sex (interaction *p*-value = 0.044)

^5^Models for waist circumference and waist-to-height ratio were not adjusted for child’s BMI

No evidence was found of interactions between screentime and the age of the participants in any of the adjusted models (*p* > 0.05). Based on variance inflation factors, there was no evidence of multi-collinearity in any of the models for both the linear and logistic regression analyses (VIF < 4). Visual inspection of residual plots against fitted values for the linear regression show that the assumptions of linearity of regression models were satisfied and the residuals seem to have constant variance without displaying any trends. In the planned sensitivity analyses, the linear and logistic regression results from complete screen time data were similar to those obtained with incomplete screen time data (data available upon request). The sensitivity analyses with and without the covariates (SSB, eating while watching TV, physical activity) showed that there was no changes in the estimates.

## Discussion

In this study, an association between daily screen time and CMR score or individual CMR factors (glucose, SBP, log triglycerides, WC, waist-to-height ratio) was not identified. After adjusting for the covariates, for every 60 min spent in front of screens, there was 0.046 mmol/L higher mean blood levels of non-HDL.

To the best of the authors’ knowledge, the present paper is one of the first studies to explore screen time and a composite measure of CMR, and a large number of individual cardiometabolic risk factors in young children under 6 years. Work by Crispim et al. [25] showed that time spent in front of the TV was not associated with elevated blood pressure. However, this study had a relatively small sample size (*n* = 276), did not adjust for other potential confounders including physical activity and family history of CVD, and did not use repeated measures of blood pressure. The results of the current study are consistent with systematic reviews that found that among children in older age groups, there was no significant association between screen time and cardiometabolic health indicators such as glucose, cholesterol, and weight status [15, 16, 62, 63]. There could be number of plausible explanations for the non-significant associations found in this study. As the age group for this study included children aged 3 to 6 years, it may be too early to see any substantial association between screen time and CMR. The use of parental reports can either underestimate or overestimate the true screen use of their child. This could be explained by factors such as recall bias, social desirability bias, or not being aware of screen-viewing behaviours [64, 65]. Parent-reported screen time is currently the only method to measure screen time behavior. A sex and SBP interaction was noted, such that increased screen time was associated with increased SBP in boys only. This finding requires further study.

### Strengths and limitations

The use of repeated measures for both the exposure and the outcome improves the estimates identified in this paper. GEES allow children with one or more measures of CMR to be included in the analysis and accounts for within-person correlation among children who had repeated measures of CMR available. The use of repeated measures also means that the present study required fewer participants to observe a significant effect. It is important to note that less than 20% of participants had repeated measures. Another strength of this work is that, in contrast to studies conducted in older age groups [8, 11, 12, 19–21, 23, 24, 26], this study focuses on all types of screen time (video games, tablets, smartphones, handheld game consoles, etc.) and includes weekend and weekday use, providing comprehensive data of screen time among this population. Looking at how the effects on CMR differ between handheld and non-handheld devices adds additional strength to this study. As screen time has changed substantially in the last 10 years including a rise in handheld devices, studies should consider how this may impact health outcomes in children. Inclusion of non-HDL as an outcome adds strength to our study as it has shown to be good predictors of future cardiometabolic health [66–68], and is the recommended measure for lipid screening in children [57, 69, 70]. Non-HDL levels during childhood are important measures of future CMR as they are associated with premature atherosclerosis, and also tracks into adulthood [71–74]. The effect size for non-HDL found in this study is small, and the clinical significance of this effect is unclear.

One limitation of this study is that as blood pressure was only measured once during the visit, we are unable to remove additional random error associated with within-person variations in blood pressure, for example, that may occur over the course of a day [75]. In this study, having screen time and cardiometabolic risk measured simultaneously, and having limited number of children with repeated measures prevents us from inferring causation. Although reverse causation cannot be ruled out, we are unaware of evidence to date that CMR would lead to a change in screen time. Additional limitations of this study include the potential for residual confounding; authors were unable to account for additional dietary factors that may contribute to cardiovascular risk, including dietary intake. Although there was missing data in some covariates, multiple imputation was used to address this limitation, and a sensitivity analysis using complete case data revealed similar findings (data not shown). The children in this study were recruited from primary care practices mainly in Toronto and Montreal, Canada, from families with higher than the average median household income and the results in this population may not be generalizable to all North American children.

## Conclusion

In this study, an association between daily screen time and total CMR score was not identified. There was a small effect between daily screen time and non-HDL cholesterol. This study serves as a first step in examining relationship between screen use and CMR factors in early childhood. As many guidelines including the Canadian Paediatric Society, the WHO and the American Academy of Paediatrics (AAP) provide screen time recommendations for children [76], it is important to evaluate the impact of screen time on health risks in early childhood outcomes. Although there is good evidence to suggest limiting screen time may improve developmental outcomes such as language development in young children, effects of screen time on cardiometabolic risk factors may not be as apparent in early childhood. Future studies are needed to assess how screen time at a very young age is related to CMR in later childhood, adolescence and adulthood.

## Supplementary information

**Additional file 1: Supplemental Table 1**. Linear GEE regression model for association between handheld screen time (h/day) and total cardiometabolic risk score and individuals risk factors, *n* = 1706. **Supplemental Table 2**. Linear GEE regression model for association between Non-handheld screen time (h/day) and total cardiometabolic risk score and individuals risk factors, n = 1706. **Supplemental Table 3**. Linear GEE regression model for association between screen time (h/day) and total cardiometabolic risk score and individuals risk factors, *n* = 1706.

## Data Availability

Available upon request.

## Abbreviations

BMI: Body Mass Index
CMR: Cardio Metabolic Risk
CVD: CardioVascular Disease
GEE: Generalized Estimating Eq.
HDL cholesterol: High-density lipoprotein cholesterol
NHQ: Nutrion and Health Questionnaire
Non-HDL cholesterol: Non-high-density lipoprotein cholesterol
SBP: Systolic Blood Pressure
SSB: Sugar-sweetened beverages
WC: Waist Circumference
