# Supplementary material for: The association between screen time and cardiometabolic risk in young children
Source: Int J Behav Nutr Phys Act. 2020 Apr 29;17:41. doi: 10.1186/s12966-020-00943-6 (PMC7189472; doi:10.1186/s12966-020-00943-6)
Supplement: Supplementary file 1 — Additional file 1: Supplemental Table 1. Linear GEE regression model for association between handheld screen time (h/day) and total cardiometabolic risk score and individuals risk factors, n = 1706. Supplemental Table 2. Linear GEE regression model for association between Non-handheld screen time (h/day) and total cardiometabolic risk score and individuals risk factors, n = 1706. Supplemental Table 3. Linear GEE regression model for association between screen time (h/day) and total cardiometabolic risk score and individuals risk factors, n = 1706. [file 12966_2020_943_MOESM1_ESM.docx]

| *Supplemental Table 1*  Linear GEE regression model for association between handheld screen time (h/day) and total cardiometabolic risk score and individuals risk factors, *n*=1,706 | | | | |  |  |  |
| --- | --- | --- | --- | --- | --- | --- | --- |
|  | **Unadjusted Model** | | **Adjusted Model^1^** | | **Adjusted (without BMI)^5^** | |  |
| **Outcome Variable** | $\hat{\beta}$ **(95% CI)** | ***p*** | $\hat{\beta}$ **(95% CI)** | ***p*** | $\hat{\beta}$ **(95% CI)** | ***p*** |  |
| Total cardiometabolic risk score | -0.159 (-0.419, 0.099) | 0.220 | -0.136 (-0.380, 0.107) | 0.264 | -0.141 (-0.403, 0.121) | 0.285 |  |
|  | | | | |  |  |  |
| Glucose (mmol/L)^2^ | -0.067 (-0.196, 0.061) | 0.299 | -0.069 (-0.204, 0.066) | 0.311 | -0.069 (-0.204, 0.066) | 0.312 |  |
| Systolic blood pressure (mmHg)^2,3^ |  |  |  |  |  |  |  |
| Females | -0.192 (-1.511, 1.128) | 0.776 | -0.610 (-1.974, 0.754) | 0.381 | -0.605 (-1.981, 0.770) | 0.388 |  |
| Males | 0.826 (-0.674, 2.326) | 0.281 | 0.124 (-1.102, 1.351) | 0.842 | 0.090 (-1.147, 1.327) | 0.887 |  |
| Triglycerides (mmol/L) (log-transformed)^2^ | -0.035 (-0.114, 0.044) | 0.384 | -0.016 (-0.095, 0.064) | 0.698 | -0.016 (-0.097, 0.064) | 0.690 |  |
| High-density lipoprotein cholesterol (mmol/L)^2^ | 0.048 (-0.017, 0.113) | 0.146 | 0.018 (-0.031, 0.067) | 0.472 | 0.018 (-0.032, 0.068) | 0.477 |  |
| Waist circumference (cm)^4^ | -0.284 (-0.815, 0.246) | 0.292 |  |  | -0.543 (-1.320, 0.235) | 0.168 |  |
| Waist-to-height ratio^4^ | -0.009 (-0.019, 0.002) | 0.098 |  |  | -0.004 (-0.010, 0.003) | 0.254 |  |
| Non-high-density lipoprotein cholesterol (mmol/L)^2^ | 0.062 (-0.054, 0.179) | 0.291 | 0.073 (-0.055, 0.202) | 0.261 | 0.073 (-0.056, 0.203) | 0.263 |  |

^1^Adjusted model includes adjustment for child age, child sex, maternal ethnicity, parental income, child’s BMI, family history of CVD, physical activity, sugar-sweetened beverages, and eating while watching TV.

^2^Model with glucose, systolic blood pressure, triglycerides, high-density lipoprotein cholesterol, and non-high-density lipoprotein cholesterol were adjusted for fasting time

^3^Models with systolic blood pressure is further adjusted for height, and include interactions for sex

^4^Models for waist circumference and waist-to-height ratio were never adjusted for child’s BMI.

^5^Partially adjusted model includes adjustment in adjusted models, except for child’s BMI.

| *Supplemental Table 2*  Linear GEE regression model for association between Non-handheld screen time (h/day) and total cardiometabolic risk score and individuals risk factors, *n*=1,706 | | | | |  |  |
| --- | --- | --- | --- | --- | --- | --- |
|  | **Unadjusted Model** | | **Adjusted Model^1^** | | **Adjusted (without BMI)^5^** | |
| **Outcome Variable** | $\hat{\beta}$ **(95% CI)** | ***p*** | $\hat{\beta}$ **(95% CI)** | ***p*** | $\hat{\beta}$ **(95% CI)** | ***p*** |
| Total cardiometabolic risk score | -0.020 (-0.078, 0.037) | 0.489 | -0.014 (-0.077, 0.048) | 0.657 | -0.009 (-0.075, 0.057) | 0.789 |
|  | | | | |  |  |
| Glucose (mmol/L)^2^ | -0.013 (-0.047, 0.020) | 0.443 | 0.002 (-0.036, 0.041) | 0.915 | 0.002 (-0.037, 0.041) | 0.918 |
| Systolic blood pressure (mmHg)^2,3^ |  |  |  |  |  |  |
| Females | -0.335 (-0.954, 0.284) | 0.289 | -0.558 (-1.220, 0.104) | 0.099 | -0.556 (-1.216, 0.104) | 0.099 |
| Males | 0.442 (-0.025, 0.908) | 0.064 | 0.166 (-0.292, 0.624) | 0.477 | 0.185 (-0.274, 0.643) | 0.430 |
| Triglycerides (mmol/L) (log-transformed)^2^ | 0.004 (-0.021, 0.028) | 0.773 | 0.012 (-0.017, 0.041) | 0.432 | 0.012 (-0.017, 0.042) | 0.412 |
| High-density lipoprotein cholesterol (mmol/L)^2^ | 0.009 (-0.005, 0.023) | 0.214 | 0.008 (-0.008, 0.023) | 0.345 | 0.007 (-0.008, 0.023) | 0.361 |
| Waist circumference (cm)^4^ | -0.078 (-0.289, 0.132) | 0.464 |  |  | -0.059 (-0.276, 0.158) | 0.593 |
| Waist-to-height ratio (cm)^4^ | -0.001 (-0.003, 0.001) | 0.178 |  |  | <0.001 (-0.002, 0.001) | 0.616 |
| Non-high-density lipoprotein cholesterol (mmol/L)^2^ | 0.036 (0.011, 0.061) | 0.005 | 0.039 (0.010, 0.069) | 0.008 | 0.040 (0.011, 0.069) | 0.007 |

^1^Adjusted model includes adjustment for child age, child sex, maternal ethnicity, parental income, child’s BMI, family history of CVD, physical activity, sugar-sweetened beverages, and eating while watching TV.

^2^Model with glucose, systolic blood pressure, triglycerides, high-density lipoprotein cholesterol, and non-high-density lipoprotein cholesterol were adjusted for fasting time

^3^Models with systolic blood pressure is further adjusted for height, and include interactions for sex

^4^Models for waist circumference and waist-to-height ratio were never adjusted for child’s BMI.

^5^Partially adjusted model includes adjustment in adjusted models, except for child’s BMI.

| *Supplemental Table 3*  Linear GEE regression model for association between screen time (h/day) and total cardiometabolic risk score and individuals risk factors, *n*=1,706 | | | | |  |  |
| --- | --- | --- | --- | --- | --- | --- |
|  | **Unadjusted Model** | | **Adjusted Model^1^** | | **Adjusted (without BMI)^5^** |  |
| **Outcome Variable** | $\hat{\beta}$ **(95% CI)** | ***p*** | $\hat{\beta}$ **(95% CI)** | ***p*** | $\hat{\beta}$ **(95% CI)** | ***p*** |
| Total cardiometabolic risk score | -0.037 (-0.086, 0.013) | 0.147 | -0.006  (-0.021 to 0.009) | 0.268 | -0.028 (-0.085, 0.030) | 0.344 |
|  | | | | |  |  |
| Glucose (mmol/L)^2^ | -0.021 (-0.050, 0.008) | 0.157 | -0.011 (-0.045, 0.023) | 0.527 | -0.011 (-0.045, 0.023) | 0.525 |
| Systolic blood pressure (mmHg)^2,3^ |  |  |  |  |  |  |
| Females | -0.518 (-1.138, 0.101) | 0.101 | -0.522 (-1.142, 0.098) | 0.099 | -0.277 (-0.843, 0.290) | 0.338 |
| Males | 0.170 (-0.243, 0.582) | 0.420 | 0.161 (-0.252, 0.573) | 0.445 | 0.480 (0.073, 0.887) | 0.021 |
| Triglycerides (mmol/L) (log-transformed)^2^ | -0.001 (-0.022, 0.020) | 0.930 | 0.008 (-0.018, 0.034) | 0.524 | 0.009 (-0.017, 0.035) | 0.508 |
| High-density lipoprotein cholesterol (mmol/L)^2^ | 0.014 (0.001, 0.026) | 0.033 | 0.008 (-0.006, 0.022) | 0.261 | 0.008 (-0.006, 0.022) | 0.274 |
| Waist circumference (cm)^4^ | -0.104 (-0.288, 0.081) | 0.271 |  |  | -0.143 (-0.336, 0.051) | 0.148 |
| Waist-to-height ratio^4^ | -0.002 (-0.004, -0.001) | 0.010 |  |  | -0.001 (-0.003, 0.001) | 0.207 |
| Non-high-density lipoprotein cholesterol (mmol/L)^2^ | 0.039 (0.015, 0.063) | 0.001 | 0.046 (0.017, 0.075) | 0.002 | 0.046 (0.017, 0.075) | 0.002 |

^1^Adjusted model includes adjustment for child age, child sex, maternal ethnicity, parental income, child’s BMI, family history of CVD, physical activity, sugar-sweetened beverages, and eating while watching TV.

^2^Model with glucose, systolic blood pressure, triglycerides, high-density lipoprotein cholesterol, and non-high-density lipoprotein cholesterol were adjusted for fasting time

^3^Models with systolic blood pressure is further adjusted for height, and include interactions for sex (interaction p-value = 0.044).

^4^Models for waist circumference and waist-to-height ratio were never adjusted for child’s BMI.

^5^Partially adjusted model includes adjustment in adjusted models, except for child’s BMI.
